# High-fidelity 3D mesh generation from a single sketch using shape constraints

**DOI:** 10.1038/s41598-025-30843-3

**Published:** 2025-12-26

**Authors:** Yingbin Wu, Fubo Wang, Peng Zhao, Mingquan Zhou, Shengling Geng, Dan Zhang

**Affiliations:** 1 School of Computer Science, Qinghai Normal University, Xining, 810016 China; 2 School of Mathematics and Information Technology, Yuncheng University, Yuncheng, 044000 China; 3 The State Key Laboratory of Tibetan Intelligence, Xining, 810016 China; 4 The Virtual Reality Application Engineering Research Center of the Ministry of Education, Beijing Normal University, Beijing, 100875 China

**Keywords:** Sketch-based generation, 3D reconstruction, Shape constrained, 3D modeling, Computer science, Computational science

## Abstract

The research on 3D model reconstruction from a single image using deep learning technology has achieved remarkable progress. However, compared with images, sketches lack sufficient visual information, which challenges the reconstruction algorithm’s ability to correctly interpret sketches. Herein, we introduce a streamlined network architecture for sketch-to-3D mesh generation, designed to address the challenge of reconstructing high-fidelity 3D models from single-hand sketches. Our approach deploys the expressive PowerMLP architecture within an encoder-decoder framework, surpassing traditional MLP implementations in representation capability. By integrating 3D shape constraints instead of relying on conventional discriminators, we achieve geometric fidelity in a collaborative generation process. Experimental results demonstrate state-of-the-art (SOTA) performance on both synthetic stylized sketches and real-world handwritten inputs, validating the method’s robustness and adaptability.

## Introduction

Driven by the rapid evolution of computer graphics and VR/AR technologies, there has been a significant increase in the demand for 3D content creation across various domains. Sketch-based 3D reconstruction has emerged as an intuitive and efficient design paradigm, attracting considerable attention from both academic and industrial communities. This approach aims to rapidly generate 3D models from simple hand-drawn sketches, thereby reducing the technical complexity associated with traditional 3D modeling techniques and addressing the growing need for 3D content in fields such as film and television animation, game development, industrial design, and architectural design^1–3^. Current methods for sketch-based 3D modeling can be broadly categorized into three groups. The first group employs 3D model retrieval techniques, which match user-provided sketches against pre-existing 3D model libraries to generate the most similar 3D shapes^3–6^. The second group leverages multi-view 2D sketches and utilizes deep neural networks to reconstruct 3D shapes directly from these sketches^7–11^. More recently, cross-modal generation techniques have gained prominence by integrating sketch-based and text-to-3D generation methods, thereby expanding the scope and flexibility of 3D content creation^12–14^.

However, these existing methods suffer from several limitations that hinder their widespread adoption. For instance, methods relying on 3D model retrieval often lack the ability to customize the retrieved models, thereby limiting the creative freedom of users. Other approaches that require multi-view sketches demand users to provide input sketches from multiple perspectives, which can be challenging for those without professional drawing skills. Similarly, methods integrating text prompts and sketch images also pose significant difficulties for non-expert users, as they necessitate both textual and visual inputs, further complicating the modeling process.

Moreover, sketches are fundamentally different from real images. Composed primarily of simple lines, sketches lack effective visual information such as texture and lighting cues, which are crucial for algorithms to accurately interpret the intended 3D geometry^15–17^. The paucity of visual cues constitutes a formidable obstacle to reliable 3D-shape inference from sketches, necessitating algorithms that are both robust and contextually aware.

**Fig. 1:**
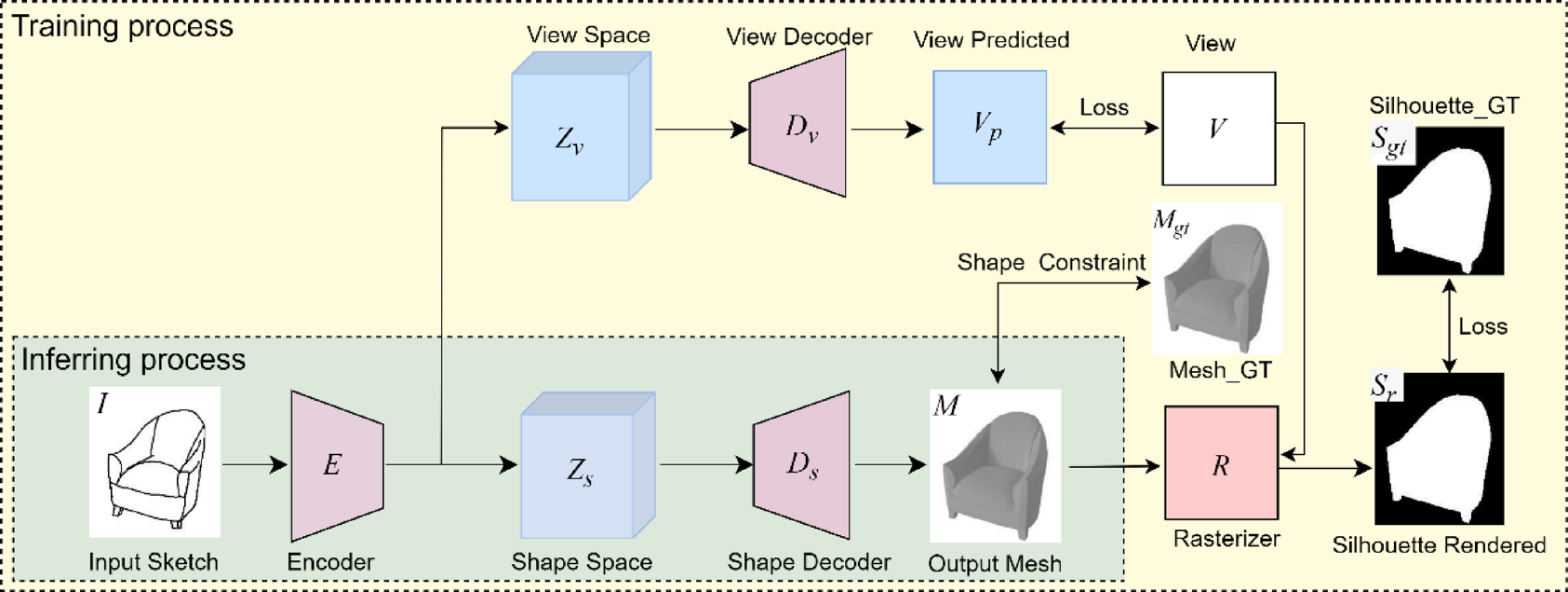
The architecture of SS2M network(The 3DViewer^18^ is used to render the 3D models displayed in the figure).

To address the aforementioned challenges, we propose a novel end-to-end neural network architecture, termed Single View Sketch-to-Mesh (SS2M), designed to provide an intuitive and efficient 3D model reconstruction framework for users. The SS2M network enables rapid and effective generation of customized, high-fidelity 3D mesh models from a single input sketch that captures the user’s conceptual intent. In the SS2M network, the input sketch is first encoded into viewpoint and shape representations within a latent space via an encoder module. Subsequently, these latent encodings are decoded into a 3D mesh model through a decoder module, as illustrated in Fig. 1. This streamlined architecture allows users to bypass the complexities associated with multi-view sketching or additional textual inputs, thereby significantly lowering the technical barriers for non-expert users while maintaining high reconstruction quality.

The SS2M network is designed as an encoder-decoder architecture with a streamlined structure. It incorporates PowerMLP^19^ neural network technology, which exhibits superior expressive capabilities compared to conventional MLP. Building upon the strengths of CNNs^20^, the SS2M encoder is capable of transforming sketch data from the input image space into a more compact and expressive low-dimensional feature representation. This enables the network to effectively capture complex shape patterns and spatial structures within sparse sketches. During the decoding process, the low-dimensional latent encoding is mapped into 3D shape data with depth information, facilitating the reconstruction of high-fidelity 3D models.

Given that traditional encoder-decoder generative networks often struggle to produce high-quality 3D shapes, and considering that discriminator-based methods^15,16^ are prone to convergence difficulties during training^21^, we introduce 3D shape constraints to replace the conventional discriminator network. This innovation serves a dual purpose: it simplifies the overall network architecture while enforcing the generation of more realistic 3D shapes by directly constraining the output of the generation network. This approach ensures that the generated 3D meshes are geometrically accurate and faithful to the input sketch, thereby enhancing the overall performance and robustness of the SS2M.

In summary, our approach has achieved superior 3D generation results through a more concise network architecture design.

Specifically, our primary contributions are summarized as follows:

1. We propose an end-to-end network model based on an encoder-decoder architecture with a streamlined structure. This model allows users to input a single-view sketch that captures their conceptual intent and rapidly generate customized, high-fidelity 3D mesh models with high efficiency.
2. To facilitate the encoding-decoding process from image space to geometric space, we introduce PowerMLP neural network technology, which exhibits stronger expressive capabilities compared to traditional MLP. This enhancement enables the SS2M network to achieve superior generation performance within a comparable training timeframe.
3. During network training, we incorporate 3D shapes from real datasets to compute similarity with the generated 3D models, serving as shape constraints to guide the network’s output. This approach enforces the generation of realistic 3D shapes, thereby improving the accuracy and plausibility of the reconstructed models.
4. Extensive experiments and evaluations conducted on Shapenet-synthetic and Shapenet-sketch datasets demonstrate that the SS2M can accurately interpret user input sketches and generate corresponding high-fidelity 3D mesh models with enhanced precision and fidelity.

## Related works

### 3D reconstruction techniques

3D model reconstruction is a vital research direction within the field of computer vision. By leveraging computational methods to reconstruct the shape, structure, texture, and other attributes of objects in three dimensions, this technology enables multi-dimensional understanding and visualization of objects. It has garnered extensive attention and found wide application across various domains, including medical health, industrial production, cultural heritage preservation, game development, and virtual reality^22,23^.

Existing 3D reconstruction technologies can be broadly categorized into three types: (1) Manual geometric modeling using software tools, which involves the creation of 3D models through manual design and manipulation in specialized software environments. (2) 3D scanning-based reconstruction, where devices such as laser radar or structured-light cameras are employed to capture the geometry of objects, and the resulting data is processed to generate 3D models. (3) Image-based 3D reconstruction, which utilizes single or multiple images and employs computer vision techniques to infer and reconstruct the 3D structure of objects from these images.

Among these approaches, image-based 3D reconstruction has gained significant popularity in practical applications due to its low cost, ease of operation, and high fidelity in reconstruction outcomes^22,23^.

### Single-view image-based 3D model reconstruction

Single-view image-based 3D object reconstruction aims to recover the 3D shape of an object from a single input image. Unlike multi-view 3D reconstruction, which leverages multiple perspectives to infer geometric information, single-view reconstruction is inherently challenging due to the limited geometric and semantic information available from a single viewpoint. The task of restoring a 3D shape from a single image is fundamentally ill-posed, as it involves significant ambiguity in depth and spatial relationships^24,25^.

Early approaches to single-view 3D reconstruction primarily relied on visual cues such as lines, shadows, and textures within the image to make geometric assumptions and reconstruct the 3D surface of the object^24,25^. However, these methods are often domain-specific and lack generalizability, resulting in poor scalability and limited applicability to diverse object classes and scenarios.

With the advent of large-scale 3D model datasets^11,26,27^ and the rapid advancement of deep learning techniques, the landscape of single-view 3D reconstruction has significantly evolved. Notably, the emergence of differentiable rendering technology in recent years^28–31^ has enabled the integration of rendering processes into end-to-end learning frameworks. This development, combined with the availability of extensive datasets, has facilitated the training of robust models capable of adapting to various complex variations in object shapes, appearances, and viewpoints^15,22,23,28,31–37^. Consequently, single-view 3D object reconstruction has become increasingly viable through large-scale data-driven approaches, paving the way for more accurate and generalizable solutions.

### Single-view sketch-based 3D model reconstruction

Single-view sketch-based 3D modeling has long been a topic of active research in the field of computer graphics and vision. Existing methods can be broadly categorized into two types: end-to-end methods and interactive methods.

Interactive methods rely on the decomposition of sketch drawing into multiple steps or the use of specific gestures^38,39^. These approaches require users to possess a certain level of strategic knowledge and expertise, which may pose challenges for non-professional users. On the other hand, end-to-end methods based on templates or retrieval can produce satisfactory results but often lack the flexibility for customization, thereby limiting their applicability in scenarios where personalized designs are desired.

In recent years, there has been a growing interest in leveraging deep learning-based end-to-end methods to directly reconstruct 3D models from sketches^5,8,12,15–17^. This approach treats the task as a single-view 3D reconstruction problem, aiming to bridge the gap between sketch inputs and 3D outputs in a more seamless and automated manner. By harnessing the power of deep neural networks, these methods have shown promise in generating high-quality 3D models directly from sketches, thereby offering a more efficient and accessible solution for users.

Sketch-based modeling is intrinsically challenging compared to traditional single-view 3D reconstruction, due to the abstraction and sparsity of sketch information. Sketches are characterized by their simplicity and lack of detailed visual cues such as texture, lighting, and shadows, which are essential for inferring 3D geometry. This scarcity of information makes the task of generating high-quality 3D shapes from sketches particularly demanding.

In this paper, we address the problem of reconstructing single-view 3D objects from single-view sketches. Building on the work in^15,28^, we propose a streamlined end-to-end neural network architecture based on an encoder-decoder framework. Our method reconstructs a 3D mesh model from a single 2D sketch of the input object by leveraging PowerMLP technology and 3D shape constraints. This integrated approach effectively improves the accuracy and plausibility of the generated 3D models.

## Method

We first establish a general baseline method for 3D model generation from a single-view sketch, as introduced in^28^. Building upon this baseline, we propose our SS2M network, which is specifically designed to produce 3D mesh model from a single-view sketch.

### Baseline

**Fig. 2:**
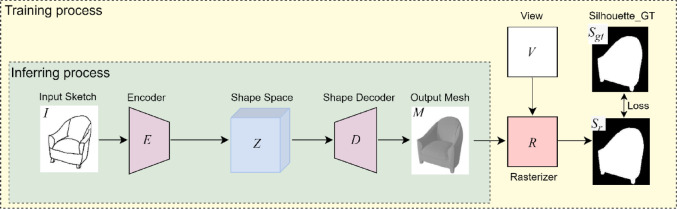
The architecture of baseline method(The 3DViewer^18^ is used to render the 3D models displayed in the figure).

As illustrated in Fig. 2, the input single sketch, denoted as *I*, is encoded into a shape code $Z=E(I)$ in the latent space via an encoder comprising the CNN^20^ and MLP. The shape code *Z* is then passed through a decoder, *D*, to generate a 3D mesh model, $M=D{\mathrm{(}}Z{\mathrm{)}}$, that closely resembles the input sketch *I*. The generated mesh model *M* is composed of vertices and faces, and can be represented as $M={\mathrm{(}}{F_M},{V_M}{\mathrm{)}}$, where ${V_M}$ denotes the vertex vector and ${F_M}$ represents the face vector in the mesh model.

During the training process, the generated mesh model *M* is rasterized by the rasterizer *R*^28^ under a specified view angle *V* to produce a rendered grayscale silhouette image ${S_r}$. This rendered image ${S_r}$ is then compared with the ground-truth rendered image ${S_{gt}}$ corresponding to the input sketch at the same view *V* to perform loss calculation. Finally, the parameters of the generator network are updated through error backpropagation to optimize the network performance.

The aforementioned process can be mathematically formulated as follows:

1 $$M=D(E(I))$$

2 $${S_r}=R(M,V)$$

It is important to note that the shape decoder *D* does not directly decode *Z* into the 3D mesh model *M*, but rather into the biases of the vertex vector ${V_M}$. This is because the output mesh *M* is constructed by deforming an isotropic sphere with a predefined set of *K*-vertices. Assuming that the vertex vector of the original *K*-vertex sphere is denoted as $B{\mathrm{(}}{F_{b}}{V_b})$, the generated mesh *M* also comprises *K* vertices. The ${F_M}$ remains as the ${F_{b}}$, but the ${V_M}$ is calculated as follows:

3 $${V_M}={V_b}+D(Z)$$

### The SS2M network architecture

The SS2M builds upon the baseline model described in Sect. 3.1 by incorporating a viewpoint prediction branch as in Sketch2Model^15^. This branch predicts the corresponding perspective information while generating the 3D model, thereby guiding the sketch generation process to determine the shape under viewpoint constraints. However, unlike Sketch2Model, we have streamlined the network architecture, eschewing additional complex branch designs in favor of a more concise structure. Furthermore, we have integrated PowerMLP^19^ technology into the encoder-decoder framework to enhance the network’s expressive capabilities.

Regarding the design of loss functions, conventional methods such as those in^15,16,37^ typically focus solely on the projection error loss between the 2D silhouette image of the generated 3D model and the input sketch. However, it is widely recognized that single-view sketches lack substantial 3D information, making it challenging to produce high-quality 3D shapes using only silhouette information for constraint and supervision. To address this limitation, we introduce a 3D shape constraint mechanism in SS2M, which directly calculates the 3D difference between the predicted shape and the ground-truth shape. This difference is incorporated as an additional term in the loss function, thereby ensuring that the generative network produces high-fidelity 3D results.

In the following section, we will elaborate on the working principle of the SS2M network in conjunction with Fig. 1.

#### Overall architecture

Assuming the input single sketch is denoted as *I*, and its corresponding output mesh is represented as $M=({F_M},{V_M})$, where ${F_M}$ and ${V_M}$ denote the face and vertex vectors of the mesh, respectively. Initially, we extract image features from the sketch *I* using the encoder *E* and map them into two separate latent spaces: the viewpoint latent space and the shape latent space. This process yields the viewpoint encoding ${Z_v}$ and the shape encoding ${Z_s}$. Mathematically, this can be expressed as:

4 $$({Z_v},{Z_s})=E(I)$$

The viewpoint decoder ${D_v}$​ decodes ${Z_v}$ into a predicted viewpoint vector ${V_p}={D_v}({Z_v})$. The loss between ${V_p}$ and the ground-truth viewpoint *V* in the sketch is then calculated. Meanwhile, the shape decoder ${D_s}$​ decodes ${Z_s}$ into a predicted 3D mesh model *M*. The vertex vector ${V_M}$ and face vector ${F_M}$ in *M* are computed as the baseline mentioned in Sect. 3.1.

The generated mesh *M* is subsequently compared with the ground-truth 3D model ${M_{gt}}$ corresponding to the sketch using a 3D shape similarity function, thereby achieving constraint control over the 3D shape fidelity. Meanwhile, under the constraint of the viewpoint *V*, the mesh *M* is rendered into a silhouette image ${S_r}=R(M,V)$ via a differentiable renderer *R*. The rendered silhouette image ${S_r}$ is then compared with the ground-truth rendered silhouette image ${S_{gt}}$ corresponding to the viewpoint *V* to compute the loss. A detailed introduction to the loss function will be provided in Sect. 3.4.

#### Encoder and decoder

The architecture of the encoder *E* is a hybrid of CNN^20^ and PowerMLP^19^, leveraging the strengths of both components. Specifically, the CNN is employed to extract salient feature information from the input sketch, focusing on essential stroke shape features while minimizing the influence of non-essential information such as painting style. Building on this foundation, two independent PowerMLP branches are utilized to map the extracted image spatial features into the viewpoint space and shape space, respectively, thereby facilitating the encoding of ${Z_v}$ and ${Z_s}$. For the decoding process, two separate PowerMLP branches are employed to decode ${Z_v}$ and ${Z_s}$ independently. Specifically, the viewpoint latent space encoding ${Z_v}$ is decoded into the viewpoint vector ${V_p}$, while the shape latent space encoding ${Z_s}$ is decoded into the vertex vector ${V_M}$ of the generated mesh *M*.

### PowerMLP

Recently, a novel neural network architecture known as the Kolmogorov Arnold Network (KAN)^40^ has garnered significant attention from researchers. Grounded in the Kolmogorov-Arnold representation theorem, KAN aims to enhance the approximation capabilities and interpretability of neural networks through an innovative mathematical design. Unlike traditional MLP, KAN introduces a unique architectural feature by placing activation functions on the edges (i.e., weights) of the network rather than on the nodes. This distinctive design enables KAN to approximate complex functions with a reduced number of parameters, which are typically parameterized using B-spline functions, thereby offering greater flexibility.

KAN has demonstrated remarkable performance in various tasks, including function fitting and solving partial differential equations. Recently, an increasing number of scholars have applied KAN or its variants to the field of computer vision, such as image and graphic classification and segmentation^19,41–44^. These applications have effectively validated the efficacy of KAN’s architecture, highlighting its potential for broader adoption in related domains.

However, the training process of KAN is significantly more complex and considerably slower than that of the traditional MLP^19,45,46^. The primary bottleneck stems from the recursive iteration process involved in calculating spline functions. In response to this challenge, PowerMLP, as a novel neural network architecture, employs a simplified representation of spline functions. By introducing a non-iterative spline function representation based on ReLU power, PowerMLP circumvents the recursive calculations required in KAN and completes the process through direct matrix operations. This approach not only retains the expressive advantages of KAN but also substantially reduces computational complexity. The *k*-th power of ReLU is defined as follows:

5 $${\sigma _k}={({\mathrm{ReLU}}({\mathrm{0}},x))^k}$$

PowerMLP is an *L*-layer neural network defined as Eq. (6):

6 $${\mathrm{PowerMLP}}(x)=({\Psi _{L - 1}} \circ \cdots \circ {\Psi _1} \circ {\Psi _0})(x)where:{\Psi _l}({x_l})=\left\{ {\begin{array}{*{20}{c}} {{\alpha _l}b({x_l}){\sigma _k}({\omega _l}{x_l}+{\gamma _l}),{\text{ for }}l<L - 1} \\ {{\omega _{L - 1}}{x_{L - 1}}+{\gamma _{L - 1}},{\text{ for }}l=L - 1} \end{array}} \right.$$

**Fig. 3:**
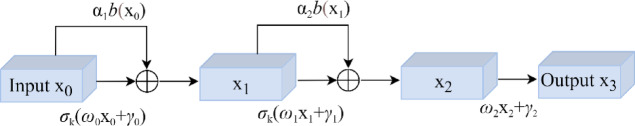
Structure of PowerMLP (3 layers).

In this context, $\alpha$, $\omega$, and $\gamma$ are all trainable arrays, ${\sigma _k}$ denotes the activation function, which is the *k*-th power of ReLU, and $b(x)$ represents the basis function, defined in the same manner as in KAN. Figure 3 illustrates a structural diagram of a PowerMLP with three layers.

Under the same parameter quantity, PowerMLP exhibits similar computational speed to traditional MLP, yet both are significantly faster than KAN. Moreover, PowerMLP demonstrates certain advantages in tasks such as machine learning. In this work, we adopt PowerMLP as the fundamental building block for the encoder and decoder in our network, replacing the conventional MLP. This choice enables more effective handling of the complex transformation from sketch image space to viewpoint and shape spaces. For detailed information regarding PowerMLP, please refer to Reference^19^.

### Shape constraint

In conventional 3D generative network models^15,16,37^, two primary approaches are typically employed for shape constraints on the generated models: (1) utilizing sketches and projected silhouettes of the generated 3D models for loss calculation to enforce shape constraints, and (2) implementing shape constraints through a GAN-based discriminator network. However, we argue that 2D projected silhouette images lack essential depth information and necessary shape and line details, thereby failing to fully represent the 3D model’s geometry. Additionally, the GAN-based discriminator network introduces unnecessary complexity to the overall network architecture.

In this work, we propose an alternative approach by employing 3D shape similarity measures as constraints. Specifically, we calculate the similarity between the generated 3D model and the ground-truth model to enforce shape constraints on the generated model. This method directly leverages 3D information, ensuring a more accurate and comprehensive representation of the model’s geometry.

In practice, common metrics for measuring the similarity between two 3D models include Chamfer Distance, Voxel IoU, Hausdorff distance, and One-sided Hausdorff distance^47,48^, among others. These metrics can be individually or jointly employed as the shape-constrained loss function ${L_{sc}}$. During training, the feedback from ${L_{sc}}$ is utilized to optimize the generative network, thereby compelling the generated meshes to exhibit realistic 3D shapes while preserving their correctness and plausibility. In this paper, the selection and definition of ${L_{sc}}$will be detailed in Sect. 3.5.

### Loss function

We have meticulously designed the loss function for training our network, which comprises several components: the progressive silhouette Intersection over Union (IoU) loss ${L_{ps}}$ between the projected silhouettes of the generated model and the ground-truth model; the shape geometric regularization loss^15,28,31^ of the generated model, which includes the Laplacian loss ${L_{lap}}$ and smoothing loss ${L_{fl}}$, the viewpoint loss ${L_v}$ between the predicted viewpoint and the ground-truth viewpoint, and the 3D shape constraint loss ${L_{sc}}$.

We first define the IoU loss ${L_{iou}}$, which quantifies the Intersection over Union between the projected silhouette ${S_r}$ of the generated model and the projected silhouette ${S_{gt}}$of the ground-truth model. The definition of ${L_{iou}}$ is as follows:

7 $${L_{iou}}=1 - \frac{{{{\left\| {{S_r} \otimes {S_{gt}}} \right\|}_1}}}{{{{\left\| {{S_r} \oplus {S_{gt}} - {S_r} \otimes {S_{gt}}} \right\|}_1}}}$$

During the training process, we employed a multi-scale, *i*-layer pyramid to render silhouette images to achieve finer rendering silhouettes. The IoU loss for the *i*-th layer silhouette within the pyramid is denoted as $L_{{iou}}^{i}$. The progressive IoU loss ${L_{ps}}$ is then defined as follows:

8 $${L_{ps}}=\sum\limits_{{i=1}}^{N} {{\lambda _{si}}L_{{iou}}^{i}}$$

In this context, *N* denotes the number of layers in the pyramid, and ${\lambda _{si}}$ is the hyperparameter associated with the IoU loss of the *i*-th layer silhouette.

The Laplacian Loss is employed to enhance the smoothness of the generated mesh. Let ${v_i}$ denote an arbitrary point within mesh *M*, and $v{'_i}$ represent the centroid of the neighboring vertices of ${v_i}$, and ${L_l}_{{ap}}$ is mathematically defined as follows:

9 $${L_{lap}}=\sum\limits_{i} {\left\| {{v_i} - v{'_i}} \right\|}$$

The Smoothing Loss minimizes the discrepancy in normal directions between adjacent triangular faces, thereby promoting the overall smoothness of the mesh surface. Specifically, the loss is formulated to penalize deviations in the normal angles between neighboring faces, where ${\theta _i}$ denotes the angle between the normal vectors of any two adjacent faces. The loss is calculated as follows:

10 $${L_{fl}}=\sum\limits_{{{\theta _i}}} {{{(\cos {\theta _i}+1)}^2}}$$

${L_v}$ calculates the loss between the predicted viewpoint and the ground-truth viewpoint. Assuming that the distance between the viewpoint and the camera is fixed, the viewpoint is represented using Euler angles. The definition is as follows:

11 $${L_v}={\left\| {V - {V_p}} \right\|_2}$$

In this work, for simplicity, we employ the Chamfer Distance as a sole measure of similarity between the generated 3D model and the ground-truth model. A smaller Chamfer Distance indicates a higher degree of similarity between the two models. We define a loss function ${L_{sc}}$ based on the Chamfer Distance to impose shape constraints on the generative model. During training, ${L_{sc}}$ leverages 3D shapes from the real dataset to compute the similarity between the generated mesh model *M* and the ground-truth 3D model ${M_{gt}}$ using the Chamfer Distance. The feedback from the ${L_{sc}}$ loss is then utilized to optimize the generative network.

The ${L_{sc}}$ loss function, defined using the Chamfer Distance, is formulated as follows:

12 $${L_{sc}}=\frac{1}{{\mathrm{n}}}({\sum\limits_{{{p_i} \in {V_M}}} {\mathop {\hbox{min} }\limits_{{{q_j} \in {V_{M\_gt}}}} \left\| {{p_i} - {q_j}} \right\|} ^2}+{\sum\limits_{{{q_j} \in {V_{M\_gt}}}} {\mathop {\hbox{min} }\limits_{{{p_i} \in {V_M}}} \left\| {{p_i} - {q_j}} \right\|} ^2})$$

In this context, ${V_M}$ and ${V_{M\_gt}}$ correspond to the vertex vectors of *M* and ${M_{gt}}$, respectively, and *n* denotes the number of vertices in the 3D mesh model.

In summary, the total loss function $Loss$ of the training network is composed of the weighted sum of the aforementioned components:

13 $$Loss={\lambda _{ps}}{L_{ps}}+{\lambda _{lap}}{L_{lap}}+{\lambda _{fl}}{L_{fl}}+{\lambda _v}{L_v}+{\lambda _{sc}}{L_{sc}}$$

## Experiments

We trained and evaluated the SS2M network on the Shapenet-synthetic dataset^11^ and further conducted hand-drawn sketch tests using the Shapenet-sketch dataset^15^ to demonstrate the effectiveness of our method. Additionally, we compared our approach with baseline methods and the state-of-the-art Sketch2Model^15^, highlighting the advantages of our proposed method through comprehensive experiments.

### Datasets

The Shapenet-synthetic dataset comprises 13 sub-datasets, each corresponding to a specific category of 3D model data, such as airplanes, cars, and tables, spanning a total of 13 categories. Each sub-dataset contains thousands of distinct instances, with each instance comprising 3D model data, 20 viewpoint data, and 2D edge (sketch) silhouette images corresponding to the rendered 3D models from these viewpoints. The Shapenet-synthetic dataset is primarily utilized for both training and testing processes.

The Shapenet-sketch dataset also consists of 13 sub-datasets corresponding to the categories in Shapenet-synthetic. Each sub-dataset contains 100 instances, with each instance comprising 3D model data, a single specified viewpoint, and the rendered image of the 3D model from that viewpoint. Moreover, for each rendered image, there is a corresponding hand - drawn sketch created by a human volunteer. This situation unavoidably leads to a domain gap, which may create a significant distinction between the rendered images and real - world visual scenes. Consequently, any 3D shape recovered from such sketches cannot be rigorously quantified against the corresponding ground-truth model; That is, if there is a significant difference between the manual sketch and the actual image, the closer the reconstruction adheres to the sketch, the larger its discrepancy with the actual 3D model becomes. The Shapenet-sketch dataset is primarily used for testing purposes.

The partitioning of the training, validation, and test sets in both the Shapenet-synthetic and Shapenet-sketch datasets is consistent with that described in^15^.

### Experimental details

#### The configuration of the experimental environment

The configuration of the experimental environment is shown in Table 1.

**Table 1:** The configuration of the experimental environment.

OS	CPU	Memory	GPU	Language	Framework
Windows	Intel Core i5-2.50 GHz	32GB	NVIDIA GeForce RTX 4080 S	Python3.10	PyTorch2.1

#### Network model configuration

For both the baseline method and SS2M, the encoder employs ResNet-18^15,20^ as the CNN to extract image features. The batch size is set to 64 and the layers of PowerMLP are 3. The Adam optimizer is utilized with a learning rate of ${l_r}=1 \times {10^{ - 4}}$, which is multiplied by 0.3 every 800 epochs. ${\beta _1}=0.9$, ${\beta _2}=0.999$. Training is conducted separately for each subclass, with a training duration of 2000 epochs. Additionally, the hyperparameters are configured as${\lambda _{ps}}=1$, ${\lambda _{lap}}=5 \times {10^{ - 3}}$, ${\lambda _{fl}}=5 \times {10^{ - 4}}$,${\lambda _v}=1$and${\lambda _{sc}}=1 \times {10^{ - 3}}$.

### Results

We evaluated the performance of SS2M by comparing it with baseline models and state-of-the-art (SOTA) models^15^ through a series of experiments.

#### Experiments with the Shapenet-synthetic dataset

We first evaluated the performance of our model using the voxel Intersection over Union metric ($Voxel\_IoU$), the calculation of which is detailed in Eq. (14)^48^.

14 $$Voxel\_IoU=\frac{{{{\left\| {M' \otimes M{'_{gt}}} \right\|}_1}}}{{{{\left\| {M' \oplus M{'_{gt}} - M' \otimes M{'_{gt}}} \right\|}_1}}}$$

Here, $M'$ and $M{'_{gt}}$ correspond to the voxelized representation of the generated mesh model *M* and the ground-truth 3D model ${M_{gt}}$, respectively.

The results are presented in Table 2.

**Table 2:** Comparisons of the mean Voxel-IoU on Shapenet-synthetic test set (Voxel-IoU↑).

	airplane	bench	cabinet	car	chair	display	lamp
Baseline	0.587	0.463	0.641	0.766	0.488	0.551	0.430
Sketch2Model(pred)	0.618	0.477	0.667	0.746	0.515	0.550	0.463
Sketch2Model(GT)	0.624	0.481	0.701	0.751	0.522	0.604	**0.472**
Ours	**0.637**	**0.519**	**0.715**	**0.789**	**0.541**	**0.617**	0.469

	loudspeaker	rifle	telephone	table	sofa	watercraft	mean
Baseline	0.622	0.610	0.681	0.513	0.616	0.565	0.579
Sketch2Model(pred)	0.624	0.606	0.673	0.470	0.620	0.569	0.584
Sketch2Model(GT)	0.641	**0.612**	0.719	**0.478**	0.622	0.586	0.601
Ours	**0.694**	0.584	**0.754**	0.452	**0.642**	**0.598**	**0.616**

As shown in Table 2, our method outperformed both the baseline and the latest existing methods, achieving the highest results in 10 out of 13 subclasses. It is important to note that Sketch2Model (pred) takes a sketch as input and predicts the viewpoint to generate the model, whereas Sketch2Model (GT) requires both the sketch and a specified viewpoint as inputs to produce the generated model. Clearly, the latter has a more complex structure (unless otherwise specified in this article, all references to Sketch2Model models refer to Sketch2Model (pred) models). Our network, by contrast, takes only a sketch as input and, overall, achieves the best average performance among the methods compared.

Additionally, we calculated the Chamfer Distance, as shown in Table 3.

**Table 3:** Comparisons of the chamfer distance on Shapenet-synthetic test set (Chamfer Distance↓).

	airplane	bench	cabinet	car	chair	display	lamp
Baseline	0.538	1.011	2.268	0.989	1.811	1.667	3.453
Sketch2Model(pred)	0.493	1.003	1.867	0.812	1.488	1.637	3.300
Sketch2Model(GT)	0.470	0.974	1.561	0.790	1.457	1.228	**3.263**
Ours	**0.469**	**0.911**	**1.548**	**0.764**	**1.431**	**1.123**	3.312

	loudspeaker	rifle	telephone	table	sofa	watercraft	mean
Baseline	2.235	**0.511**	1.268	**1.687**	2.203	0.885	1.579
Sketch2Model(pred)	2.021	0.604	1.120	1.790	1.944	1.018	1.469
Sketch2Model(GT)	1.884	0.574	0.928	1.744	2.011	0.897	1.368
Ours	**1.765**	0.613	**0.874**	1.803	**1.824**	**0.785**	**1.325**

As shown in Table 3, our method also achieved the best average performance overall.

Moreover, we quantified the standard deviation(SD) across all samples within each category (Tables 4 and 5), thereby corroborating the robustness of SS2M.

**Table 4:** Comparisons of the SD for the mean Voxel-IoU (SD↓).

	airplane	bench	cabinet	car	chair	display	lamp
Sketch2Model(pred)	0.15	0.153	0.22	0.125	0.156	0.197	0.21
Ours	**0.113**	**0.122**	**0.163**	**0.095**	**0.146**	**0.135**	**0.121**

	loudspeaker	rifle	telephone	table	sofa	watercraft	**mean**
Sketch2Model(pred)	0.216	0.172	0.216	0.181	0.162	0.168	0.179
Ours	**0.174**	**0.161**	**0.166**	**0.143**	**0.123**	**0.114**	**0.137**

As shown in Table 4, SS2M also achieves the highest overall mean SD performance in terms of voxel IoU.

**Table 5:** Comparisons of the SD for chamfer distance (SD↓).

	airplane	bench	cabinet	car	chair	display	lamp
Sketch2Model(pred)	0.181	0.339	0.631	0.118	0.406	0.652	2.031
Ours	**0.121**	**0.223**	**0.558**	**0.112**	**0.356**	**0.561**	**1.994**

	loudspeaker	rifle	telephone	table	sofa	watercraft	mean
Sketch2Model(pred)	0.583	0.347	0.480	**0.627**	0.623	0.375	0.569
Ours	**0.436**	**0.331**	**0.368**	0.672	**0.475**	**0.312**	**0.501**

As shown in Table 5, SS2M likewise attains the highest overall mean SD performance on Chamfer Distance. As evidenced by the SD metrics reported in Tables 4 and 5, the proposed model exhibits superior generalization capacity and robustness compared with the baselines.

#### The visual results obtained on the Shapenet-synthetic dataset

**Fig. 4:**
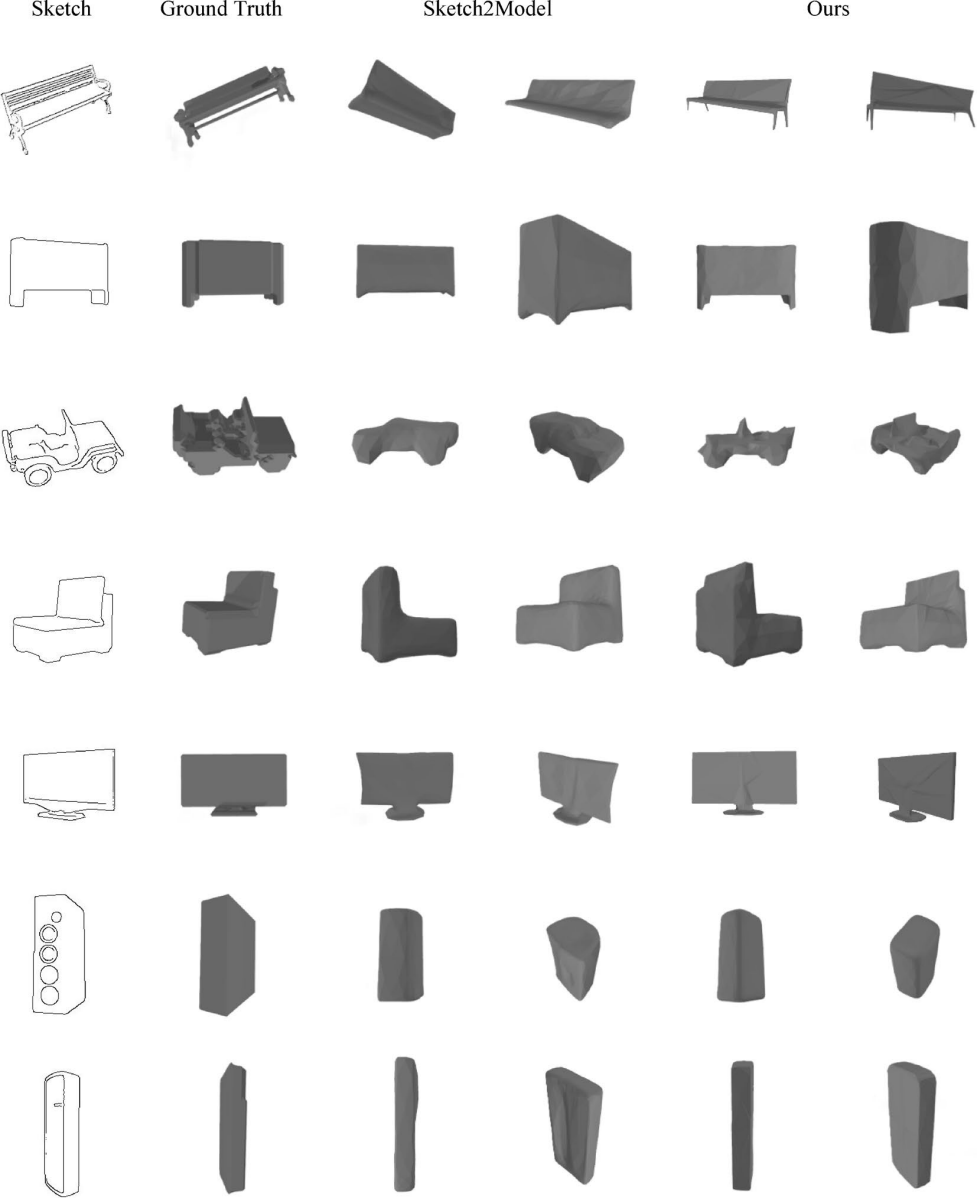
Selected representative results on the Shapenet-synthetic Dataset(The 3DViewer^18^ is used to render the 3D models displayed in the figure).

We further selected representative experimental results illustrated in Figs. 3, 4 and compared them with the latest existing technologies. Overall, the models generated by our method are visually closer to the shapes of real 3D objects, thereby further demonstrating the effectiveness of our approach in generating models with higher structural quality and fidelity.

#### The visual results obtained on the Shapenet-sketch dataset

**Fig. 5:**
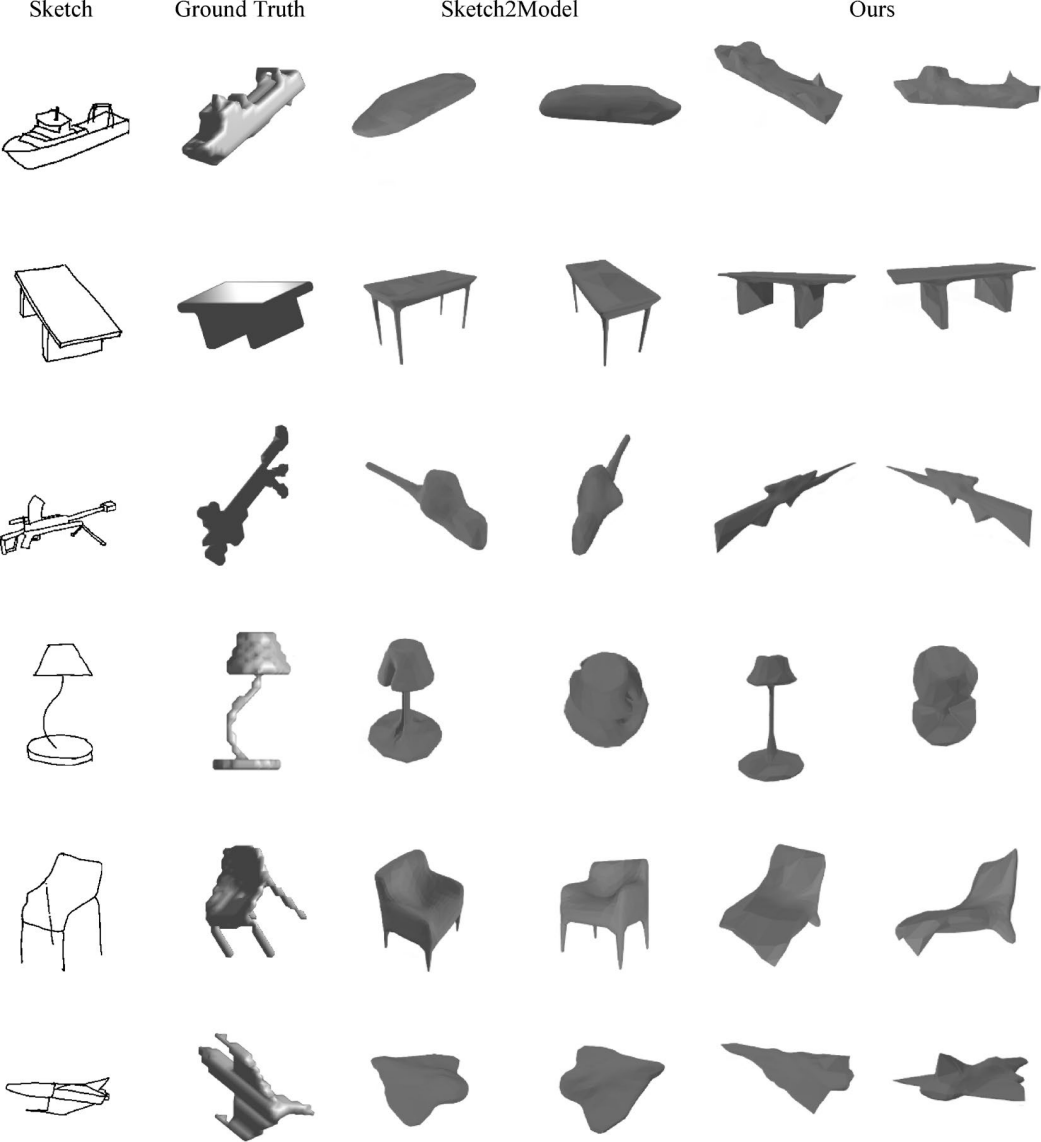
Selected representative results on the Shapenet-sketch Dataset(The 3DViewer^18^ is used to render the 3D models displayed in the figure).

Additionally, we sampled a representative subset of hand-drawn sketches from the Shapenet-sketch corpus and contrasted the reconstructions generated by our framework with those of Sketch2Model. As evidenced in Fig. 5, our SS2M consistently produces silhouettes that align more closely with the input sketches and recovers overall geometries that more faithfully encode the draftsperson’s intent, furnishing further empirical support for the superiority of the proposed method.

#### Assessing the runtime performance of 3D modeling

As previously described, our SS2M network adopts a structurally simplified encoder-decoder architecture. We evaluated the inference speed of the network on a PC equipped with an NVIDIA GeForce RTX 4080 S GPU. Our method achieved an average generation speed of 0.021 s, which is 33.3% faster than Sketch2Model (0.028 s). This result further demonstrates the advantages of our method in terms of network structure and computational efficiency.

### Ablation study

We conducted ablation studies to validate the effectiveness of the SS2M network structure by calculating the Voxel Intersection over Union (Voxel-IoU) index, with the results presented in Table 6. The findings demonstrate that, compared to the baseline method, the incorporation of PowerMLP (PM) and shape constraints (SC) significantly enhanced the performance of the generative network, as shown in Table 6.

**Table 6:** Ablation study on Shapenet-synthetic test set(Voxel-IoU↑).

PM	SC	airplane	bench	cabinet	car	chair	display	lamp
		0.587	0.463	0.641	0.766	0.488	0.551	0.430
√		0.606	0.494	0.701	0.781	0.491	0.595	**0.471**
√	√	**0.637**	**0.519**	**0.715**	**0.789**	**0.541**	**0.617**	0.469

		loudspeaker	rifle	telephone	table	sofa	watercraft	mean
		0.622	0.610	0.681	0.513	0.616	0.565	0.579
√		0.654	**0.621**	0.741	**0.515**	0.627	0.577	0.606
√	√	**0.694**	0.584	**0.754**	0.452	**0.642**	**0.598**	**0.616**

**Fig. 6:**
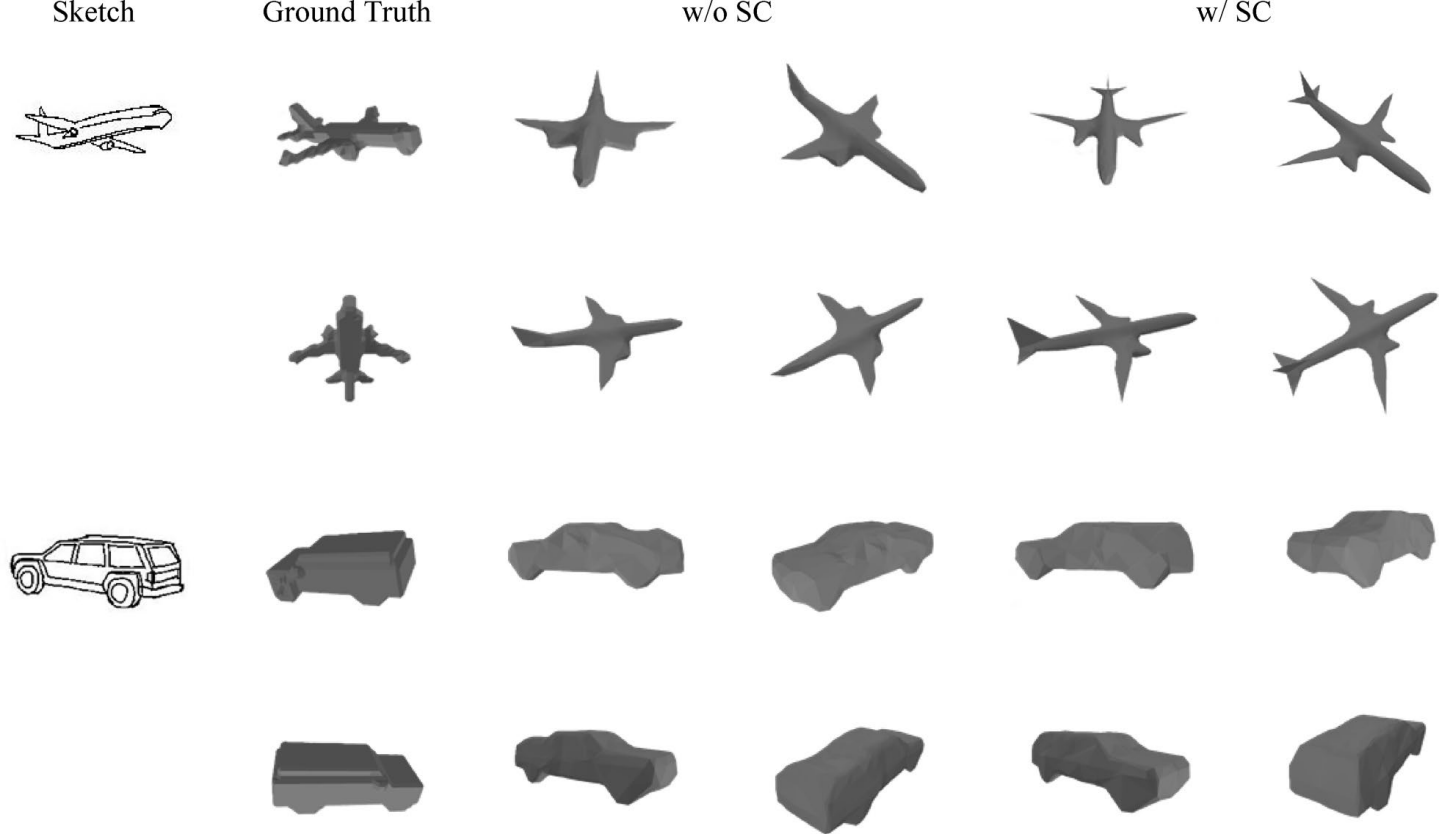
Experimental results of shape constraint effectiveness on Shapenet-sketch dataset(The 3DViewer^18^ is used to render the 3D models displayed in the figure).

We further illustrate that, compared to the absence of shape constraints (SC), our method, which incorporates shape constraints, can generate models with higher fidelity, as demonstrated in Fig. 6. For instance, in Fig. 6, the tail wing and engine components of the aircraft, as well as the rear area of the car, have been accurately reconstructed under the guidance of shape constraints.

## Conclusion

We introduce SS2M, a streamlined sketch-based 3D mesh generation network that requires only a single sketch as input to produce high-fidelity 3D mesh models. By integrating PowerMLP into the encoder-decoder architecture, our model capitalizes on its superior expressive capabilities to achieve more accurate reconstructions. Additionally, we replace traditional discriminator networks with 3D shape constraints to directly control the shape of the generated 3D models, thereby enhancing the fidelity of the reconstructed meshes. Extensive experiments on both the Shapenet-synthetic and Shapenet-sketch datasets demonstrate that our method achieves state-of-the-art (SOTA) performance for both synthesized and hand-drawn sketches. Moreover, ablation studies validate the effectiveness of the key components in our proposed approach.

In this study, we employed an early ResNet architecture for feature extraction of the sketches. Future research may benefit from incorporating state-of-the-art image feature extractors, such as the recently introduced Mamba framework^49,50^, to further enhance the fidelity of the synthesized models. Moreover, the recent emergence of generative AI, most notably diffusion models^51,52^, has advanced to the point where their integration into sketch-to-3D pipelines for stochastic 3D variant generation is now a viable and promising research avenue. Furthermore, given our emphasis on sketch-based 3D reconstruction within known categories, we note that existing 3D benchmarks derived from Shapenet remain limited in both sample size and categorical coverage. Future work will therefore investigate category-agnostic 3D object generation networks with enhanced generalization capabilities. Concurrent advances in federated learning frameworks^53,54^ and industrial metaverse infrastructures^55,56^ offer a privacy-preserving, regulation-compliant mechanism for multi-party collaboration. By orchestrating distributed training, inference and optimization within these secure environments, we can (i) expand the semantic and stylistic expressiveness of generative models across heterogeneous sketch domains and (ii) systematically enhance the geometric fidelity and plausibility of sketch-driven 3D reconstruction.

## Data Availability

The Shapenet Datasets are available at: https://shapenet.org. Further data will be made available from the corresponding author on reasonable request.
